# Treatment- and immune-related adverse events of immune checkpoint inhibitors in advanced lung cancer

**DOI:** 10.1042/BSR20192347

**Published:** 2020-05-07

**Authors:** Jun Shao, Chengdi Wang, Pengwei Ren, Yuting Jiang, Panwen Tian, Weimin Li

**Affiliations:** 1 Department of Respiratory and Critical Care Medicine, West China Medical School/West China Hospital, Sichuan University, Chengdu, China; 2 Department of Clinical Research Center for Respiratory Diseases, West China Hospital, Sichuan University, Chengdu, Sichuan, China; 3 West China Medical School, Sichuan University, Chengdu, China

**Keywords:** adverse event, chemotherapy, Immune checkpoint inhibitor, lung cancer

## Abstract

Background: Immune checkpoint inhibitors (ICIs) emerged as the preferred therapy in advanced lung cancer, understanding the treatment- and immune-related adverse events of these drugs is of great significance for clinical practice.

Materials and methods: PubMed, Embase, Cochrane library and major conference proceedings were systematically searched for all randomized controlled trials (RCTs) in lung cancer using PD-1/PD-L1/CTLA-4 inhibitors. The outcomes included treatment-related adverse events (TRAEs) and several organ specific immune-related adverse events (IRAEs).

Results: 24 RCTs involving 14,256 patients were included. There was a significant difference for ICI therapy in the incidence of any grade of TRAEs (RR: 0.90; 95%CI: 0.84–0.95; *P*=0.001) and a lower frequency of grade 3-5 of TRAEs (RR: 0.65; 95%CI: 0.51–0.82; *P*<0.001). Patients treated with ICI therapy in non–small-cell lung cancer (NSCLC) were less reported TRAEs than in small cell lung cancer (SCLC). A lower risk of TRAEs was favored by anti-PD-1 inhibitors over anti-PD-L1 antibodies and anti-CTLA-4 drugs. The most common organ specific IRAE was hypothyroidism that occurred 8.7%. The incidence of pneumonitis and hepatitis reached 4.5% and 4.0% respectively. Compared with patients treated in control arms, those treated with ICI drugs were at higher risk for each organ specific adverse event including colitis, hepatitis, pneumonitis, hypothyroidism and hypophysitis.

Conclusions: ICI therapy was safer than chemotherapy, especially ICI monotherapy such as anti-PD-1 antibodies in NSCLC. Compared with standard treatments, ICI drugs increased the risk of organ-specific IRAEs, although the overall incidence remained low.

## Introduction

Lung cancer still remains the most commonly diagnosed carcinoma type and the leading cause of cancer death globally [1]. According to the GLOBCAN report, an estimated 2.09 million new cases were diagnosed in 2018 [2]. Non–small-cell lung cancer (NSCLC) represents 85% of all lung tumors, and the other 15% is small-cell lung cancer (SCLC) [3]. Approximately one-third of patients with NSCLC have locally advanced disease at diagnosis [4]. Conventional therapy standard of first-line care treatment for advanced NSCLC and extensive-stage small-cell lung cancer (ES-SCLC) is platinum-doublet chemotherapy [5]. Despite over 30 years of clinical research, little progress has been made, and outcome of lung cancer remains poor [6]. Even in the most recent large randomized clinical trials (RCTs), the median overall survival (OS) of metastatic SCLC patients receiving standardized chemotherapy was still between 9 and 11 months [7–9]. The discovery of anaplastic lymphoma kinase (ALK) gene rearrangement in NSCLC in 2007 led to an understanding of its significance of disease biology and natural history [10]. Subsequently, the targetable genetic alterations of lung cancer have been gradually identified such as epidermal growth factor receptor (EGFR) mutations, kirsten rat sarcoma (KRAS) mutations and rat osteosarcoma (ROS1), and the development of targeted drugs greatly affected the prognosis of patients [11,12]. However, only a small proportion of patients harbor these mutations, and targeted drug therapy did not significantly improve 5-year overall survival of lung cancer patients [13].

The rapid development of immune checkpoint inhibitor (ICI), a revolutionary form of immunotherapy, has transformed the way numerous cancer are managed [14]. Inhibitory checkpoint molecules produced during T-cell activation, such as cytotoxic T-lymphocyte-associated protein 4 (CTLA-4) that regulates the immune synapses between T cells and lymph node dendritic cells to inhibit T-cell activation, or programmed death 1 (PD-1)/programmed death ligand 1 (PD-L1) suppressing the immune synapses between T cells and tumor cells, are currently the most relevant targets for immunotherapy [15]. In 2011, Food and Drug Administration (FDA) approved the first checkpoint inhibitor ipilimumab, which is a fully human anti-CTLA-4 monoclonal antibody. Later, several immune checkpoint inhibitors directed at PD-1 (nivolumab and pembrolizumab) and PD-L1 (atezolizumab, durvalumab and avelumab) were approved for the treatment of multiple cancers [16,17]. These drugs improved clinical survival outcomes of solid cancer such as lung cancer dramatically. In a review of the literature, pembrolizumab has showed a significant survival benefit over chemotherapy when given as monotherapy or as part of combination therapy for metastatic, squamous or non-squamous NSCLC [18–20].

Like chemotherapy, immunotherapy can have serious treatment-related adverse events (TRAEs) leading to low compliance, dose reduction, delayed treatment or treatment rejection, although some studies illustrated anti-PD-1 drugs were overall less toxic than standard chemotherapy [21–23]. At the same time, ICI drugs have immune-related adverse events (IRAEs) reported on clinical trials. The IRAEs including colitis, hepatitis, pneumonitis, hypothyroidism, hyperthyroidism and so on affect multiple organ systems including skin, colon, endocrine organs, liver and lungs [24]. Here, we performed a systematic and meta-analysis of immunotherapy safety. High quality studies focusing on adverse events are required to aid clinicians to improve early management and identify IRAEs.

## Materials and methods

### Search strategy

A literature search of studies published up to March 2019 was performed from major citation databases, including PubMed, Embase and Cochrane Library. The following search terms were used: immune checkpoint inhibitor, PD-1 or programmed death 1, PD-L1 or programmed death ligand 1, CTLA-4 or cytotoxic T-lymphocyte-associated protein 4, nivolumab, pembrolizumab, atezolizumab, avelumab, durvalumab, ipilimumab, or tremelimumab, and lung cancer, randomized controlled trial. To identify additional studies, we also searched the major international congresses’ proceedings (American Society of Clinical Oncology, the European Society of Medical Oncology and the World Conference of Lung Cancer). When duplicate publications were identified, the most recent, relevant and comprehensive data were accepted. The present study was carried out according to the Preferred Reporting Items for Systematic Reviews and Meta-Analysis (PRISMA) statement [25].

### Study selection

Trails were eligible for inclusion if they met several criteria: (1) patients were pathologically diagnosed with lung cancer; (2) studies involving participants treated with ICI or ICI plus chemotherapy; (3) trails which the control was chemotherapy alone; (4) main outcome was treatment-related adverse events of any grade and grade 3-5; (5) phase II or III randomized controlled trials. Studies were exclusion: (1) retrospective or prospective cohort studies; (2) reviews, letters, commentaries, irrelevant abstract, quality of life studies, cost effectiveness analyses; (3) publications without detailed safety data.

### Data extraction and quality assessment

Two investigators independently extracted data from each study with a piloted collection form: name of first author, year, trial phases, study ID, region, trial phase, types of tumor, treatment, the size of intervention and control group, TRAEs reporting rate, the frequency of specific adverse event and median follow-up time. The risk of bias was assessed by using Cochrane Risk of Bias Tool [26]. This scale evaluates six criteria: randomized sequence generation; allocation concealment; blinding of participants and personnel; blinding of outcome assessment; incomplete outcome data; selective reporting, and other bias. Each aspect was labeled as high, low or unclear risk. All disagreements in the study selection, data extraction and quality assessment were resolved by consensus.

Our primary outcome was the incidence of TRAEs, which indicated the toxicity of therapy. Our secondary outcome was the incidence of commonly described organ specific adverse events (colitis, hepatitis, pneumonitis, hypothyroidism and hypophysitis). We recorded data from full article and supplementary appendix. Common terms classified by clinical adverse events (CTCAE) were used to identify grade 3-5 as serious and grade 1-2 as other. Data from different dosing arms within the same study were extracted and reported separately.

### Statistical analysis

For each of the included studies, we calculated the odds ratio and 95% confidence interval of event incidence between the intervention group and the control group based on the number of reported events and sample size. Risk ratio (RR) and 95% confidence interval (CI) were pooled to quantify the therapeutic effect. The heterogeneity of effect size estimates across studies was described with the *I*^2^ index and *Q* statistic’s *P* value. If significant heterogeneity was not present (*P*>0.1), the risk ratio was calculated with fixed effect meta-analysis; otherwise, a random effects model was applied to calculate pooled odds ratio and 95% confidence interval if significant heterogeneity was present (*P*≤0.1). We used funnel plots to assess publication bias. Two-sided *P* values less than 0.05 were considered statistically significant. All statistical analyses were conducted using Stata version 15.0 (StataCorp, College Station, TX).

## Results

### Eligible studies and characteristics

**Figure 1:**
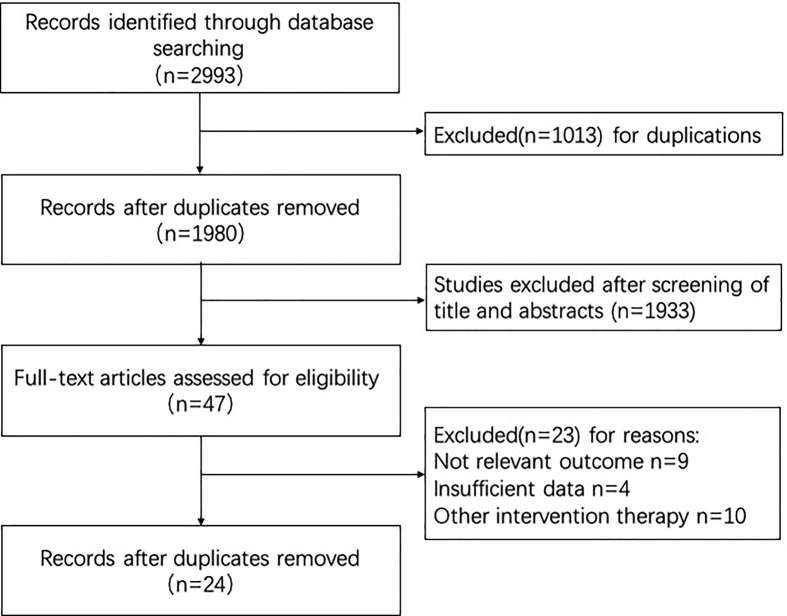
Flow chart of study selection and design/study flow diagram

A total of 2993 records were initially in line based on the literature search, of which 1013 excluded because of duplications. After screening the titles, abstracts, full article, 24 randomized controlled trials (RCTs) were finally identified in strict inclusion and exclusion criteria. Data were obtained from published manuscripts and conference proceedings. The selection process was presented in Figure 1.

All 24 studies included 14,256 patients representing advanced lung cancer were international multi center studies [8,18–20,27–46]. Twenty-one studies evaluated NSCLC, and the other three studies investigated ES-SCLC. About 7613 patients who received ICI monotherapy or combination therapy served as the investigational arm and 6643 patients who received chemotherapy as the control arms. KEYNOTE010 that analyzed two different doses (2 and 10 mg/kg) compared with standard control was divided into two trails. All grades, grade 3 and grade 4 adverse events indicate complete, severe and life threatening toxicity, respectively. The main characteristics of the included studies are summarized in Table 1.

**Table 1:** Characteristics of patients comparing ICI therapy with Chemotherapy in included randomized controlled trials

First author	Study ID	Trial Phase	Cancer Type	Treatment	ICI drug	NO OF Patients	TRAEs all grade	TRAEs grade 3-5
Borghaei, 2018	KEYNOTE021	II	NSCLC	Pembrolizumab+Chemotherapy	PD-1	55	24	59
				Chemotherapy		57	17	62
Gandhi, 2018	KEYNOTE189	III	NSCLC	Pembrolizumab+Chemotherapy	PD-1	404	272	405
				Chemotherapy		200	133	202
Paz-Ares, 2018	KEYNOTE407	III	NSCLC	Pembrolizumab+Chemotherapy	PD-1	273	194	278
				Chemotherapy		274	191	280
Herbst, 2016	KEYNOTE010	II/III	NSCLC	Pembrolizumab, 2 mg/kg	PD-1	215	43	339
				Pembrolizumab, 10 mg/kg	PD-1	226	55	343
				Chemotherapy		251	109	309
Reck, 2016	KEYNOTE024	III	NSCLC	Pembrolizumab	PD-1	113	41	154
				Chemotherapy		135	80	150
Lopes, 2018	KEYNOTE042	III	NSCLC	Pembrolizumab	PD-1	399	113	636
				Chemotherapy		553	252	615
Borghaei, 2018	CheckMate227(a)	III	NSCLC	Nivolumab+Chemotherapy	PD-1	158	89	172
				Chemotherapy		141	64	183
Hellmann, 2018	CheckMate227(b)	III	NSCLC	Nivolumab	PD-1	42	30	391
				Chemotherapy		79	61	570
Brahmer, 2015	CheckMate017	III	NSCLC	Nivolumab	PD-1	76	9	131
				Chemotherapy		111	71	129
Carbone, 2017	CheckMate026	III	NSCLC	Nivolumab	PD-1	190	47	267
				Chemotherapy		243	133	263
Borghaei, 2015	CheckMate057	III	NSCLC	Nivolumab	PD-1	199	30	287
				Chemotherapy		236	144	268
Wu, 2019	CheckMate078	III	NSCLC	Nivolumab	PD-1	216	35	337
				Chemotherapy		130	74	156
Jotte, 2018	IMpower131	III	NSCLC	Atezolizumab+Chemotherapy	PD-L1	316	231	334
				Chemotherapy		303	193	334
Papadimitrakopoulou, 2018	IMpower132	III	NSCLC	Atezolizumab+Chemotherapy	PD-L1	267	167	291
				Chemotherapy		239	114	274
Horn, 2018	IMpower133	III	ES-SCLC	Atezolizumab+Chemotherapy	PD-L1	188	115	198
				Chemotherapy		181	113	196
Socinski, 2018	IMpower150	III	NSCLC	Atezolizumab+Chemotherapy	PD-L1	371	230	393
				Chemotherapy		376	197	394
Rittmeyer, 2017	OAK	III	NSCLC	Atezolizumab	PD-L1	390	90	609
				Chemotherapy		496	247	578
Fehrenbacher, 2016	POPLAR	II	NSCLC	Atezolizumab	PD-L1	95	17	142
				Chemotherapy		119	55	135
Barlesi, 2018	JAVELIN Lung 200	III	NSCLC	Avelumab	PD-L1	251	39	393
				Chemotherapy		313	180	365
Antonia, 2018	PACIFIC	III	NSCLC	Durvalumab+Chemoradiotherapy	PD-L1	460	142	475
				Chemotherapy		222	61	234
Lynch, 2012	CA184-041(a)	II	NSCLC	Ipilimumab+Chemotherapy	CTLA-4	54	29	71
				Chemotherapy		52	24	65
Reck, 2013	CA184-041(b)	II	ES-SCLC	Ipilimumab+Chemotherapy	CTLA-4	35	18	42
				Chemotherapy		40	13	44
Govindan, 2017	CA184-104	III	NSCLC	Ipilimumab+Chemotherapy	CTLA-4	344	205	388
				Chemotherapy		292	129	361
Reck, 2016	CA184-156	III	ES-SCLC	Ipilimumab+Chemotherapy	CTLA-4	391	231	478
				Chemotherapy		361	214	476

**Abbreviations:** CTLA-4, cytotoxic T-lymphocyte-associated protein 4; ES-SCLC, extensive-stage small cell lung cancer; ICI, immune checkpoint inhibitors; NSCLC: non–small cell lung cancer; PD-1, programmed death 1; PD-L1, programmed death ligand 1; TRAE, treatment-related adverse event.

### Treatment-related adverse event

**Figure 2:**
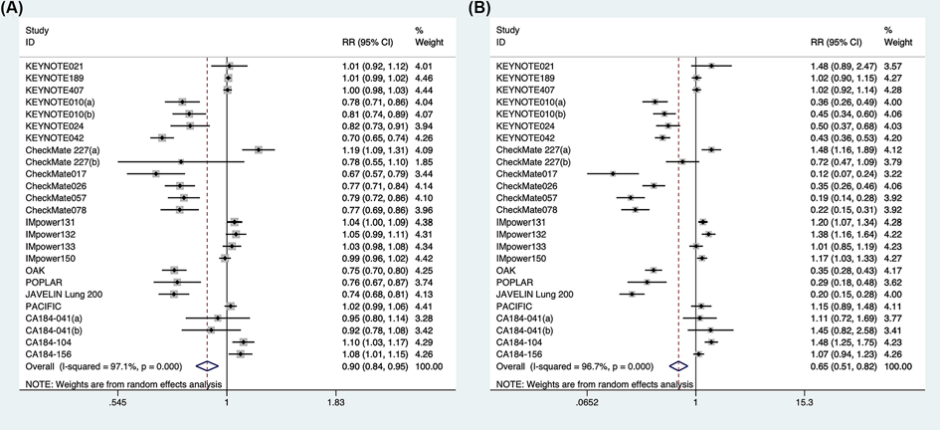
Forest plot of all grade (**A**) and grade 3-5 (**B**) TRAEs in lung cancer patients treated with ICI versus chemotherapy

In terms of ICI therapy in advanced lung cancer, there was a significant difference in the probability of any grade of TRAEs (RR: 0.90; 95%CI: 0.84–0.95; *P*=0.001) and a lower frequency of grade 3-5 of TRAEs (RR: 0.65; 95%CI: 0.51–0.82; *P*<0.001) (Figure 2). However, subgroup analysis demonstrated ICI-chemotherapy associated with the risk of TRAEs (any grade: RR: 1.03; 95%CI: 1.01–1.06; *P*=0.017; grade 3-5: RR: 1.18; 95%CI: 1.09–1.28; *P*<0.001). ICI monotherapy was safer in the risk of grade 1-5 (RR: 0.76; 95%CI: 0.73–0.78; *P*<0.001)and grade 3-5 (RR: 0.33; 95%CI: 0.26–0.41; *P*<0.001) adverse events than standard control. The finding indicated that ICI therapy led to a significant difference in NSCLC for TRAEs (any grade: RR: 0.88; 95%CI: 0.82–0.95; *P*=0.001; grade 3-5: RR: 0.60; 95%CI: 0.46–0.78; *P*<0.001), but no statistical significance in SCLC (any grade: RR: 1.03; 95%CI: 0.97–1.10; *P*=0.318; grade 3-5: RR: 1.06; 95%CI: 0.95–1.18; *P*=0.280). A lower risk of any grade (RR: 0.85; 95%CI: 0.74–0.97; *P*=0.018) or grade 3-5 (RR: 0.50; 95%CI: 0.35–0.73; *P*<0.001) adverse events was favored by anti-PD-1 antibodies over anti-PD-L1 antibodies (any grade: RR: 0.92; 95%CI: 0.83–1.01; *P*=0.090; grade 3-5: RR: 0.70; 95%CI: 0.46–1.05; *P*=0.086). Anti-CTLA-4 antibodies was not different from conventional therapy in any grade TRAEs (RR: 1.05; 95%CI: 0.98–1.12; *P*=0.179), but less safe in grade 3-5 (RR: 1.25; 95%CI: 1.00–1.55; *P*=0.047; Table 2).

**Table 2:** Risk ratios for treatment-related adverse events (TRAEs) comparing ICI therapy with Chemotherapy

All grade						Grade 3-5					
	No. of trials	No. of patients	*I*^2^ (P)	RR (95%CI)	*P*		No. of trials	No. of patients	*I*^2^ (P)	RR (95%CI)	*P*
Overall*	25	14256	97.1% (<0.001)	0.90 (0.84,0.95)	0.001	**Overall**	25	14256	96.7% (<0.001)	0.65 (0.52,0.82)	<0.001
**Subgroup**						**Subgroup**					
**Method**						**Method**					
** ICI-Chem**	13	6689	77.4% (<0.001)	1.03 (1.01,1.06)	0.017	**ICI-Chem**	13	6689	61.8% (0.002)	1.18 (1.09,1.28)	<0.001
** ICI**	12	7567	19.0% (0.257)	0.76 (0.73,0.78)	<0.001	**ICI**	12	7567	82.9% (<0.001)	0.33 (0.26,0.41)	<0.001
**Cancer Type**						**Cancer Type**					
**NSCLC**	22	12822	97.8% (<0.001)	0.88 (0.82,0.95)	0.001	**NSCLC**	22	12822	97.1% (<0.001)	0.60 (0.46,0.78)	<0.001
**SCLC**	3	1434	50.0% (0.135)	1.03 (0.97,1.10)	0.318	**SCLC**	3	1434	0.0% (0.460)	1.06 (0.95,1.18)	0.280
**ICI drug**						**ICI drug**					
**Anti-PD-1**	13	6986	98.8% (<0.001)	0.85 (0.74,0.97)	0.018	**Anti-PD-1**	13	6986	96.8% (<0.001)	0.50 (0.35,0.73)	<0.001
**Anti-PD-L1**	8	5345	96.7% (<0.001)	0.92 (0.83,1.01)	0.090	**Anti-PD-L1**	8	5345	97.5% (<0.001)	0.70 (0.46,1.05)	0.086
**Anti-CTLA-4**	4	1925	47.6% (0.126)	1.05 (0.98,1.12)	0.179	**Anti-CTLA-4**	4	1925	66.6% (0.030)	1.25 (1.00,1.55)	0.047

**Abbreviations**: CI, confidence interval; RR, risk ratio.

### Immune-related adverse event

Among intervention group, the most common IRAE was hypothyroidism that occurred 8.7%, while colitis, hepatitis, pneumonitis and hypophysitis occurred 1.6%, 4.0%, 4.5% and 0.6% respectively. Looking at serious organ specific IRAEs, 1.8% patients had hepatitis, 1.5% patients with pneumonitis, 0.8% patients with colitis, 0.3% patients with hypothyroidism and 0.3% patients had hypophysitis. Table 3 shows the rates of organ specific serious immune-related adverse events.

**Table 3:** Incidence of organ specific immune-related adverse events (IRAEs); value are percentage (95% confidence intervals)

Author	Study ID	Intervention	Colitis		Hepatitis		Pneumonitis		Hypothyroidism		Hypophysitis	
			All	Serious	All	Serious	All	Serious	All	Serious	All	Serious
Borghaei, 2018	KEYNOTE021	59	1.7	0.0	NA	NA	6.8	1.7	15.3	0.0	NA	NA
Gandhi, 2018	KEYNOTE189	405	2.2	0.7	1.2	1.0	4.4	2.7	6.7	0.5	0.7	0.0
Paz-Ares, 2018	KEYNOTE407	278	2.5	2.2	1.8	1.8	6.5	2.5	7.9	0.4	1.1	0.7
Herbst, 2016	KEYNOTE010(a)	339	1.2	0.9	NA	NA	4.7	2.1	8.3	0.0	0.3	0.3
Herbst, 2016	KEYNOTE010(b)	343	0.6	0.3	NA	NA	4.4	2.0	8.2	0.0	0.3	0.3
Reck, 2016	KEYNOTE024	154	1.9	1.3	NA	NA	5.8	2.6	9.1	0.0	0.6	0.6
Wu, 2019	CheckMate078	337	NA	NA	NA	NA	3.0	1.2	NA	NA	NA	NA
Jotte, 2018	IMpower131	334	1.8	1.2	17.4	5.4	6.9	1.2	10.2	0.6	NA	NA
Papadimitrakopoulou, 2018	IMpower132	291	1.7	0.7	4.5	2.4	5.5	2.1	7.9	0.7	NA	NA
Horn, 2018	IMpower133	198	1.5	1.0	7.1	3.5	2.0	0.5	12.6	0.0	0.5	0.0
Socinski, 2018	IMpower150	393	2.3	1.3	2.0	1.0	2.8	1.3	12.7	0.3	0.8	0.3
Rittmeyer, 2017	OAK	609	0.3	0.0	0.3	0.3	1.0	0.7	NA	NA	NA	NA
Fehrenbacher, 2016	POPLAR	142	1.4	0.7	0.7	0.0	2.8	0.7	5.6	0.7	NA	NA
Barlesi, 2018	JAVELIN Lung 200	393	0.3	NA	NA	NA	2.3	NA	4.8	NA	NA	NA
Antonia, 2018	PACIFIC	475	NA	NA	NA	NA	10.7	1.7	9.3	0.2	NA	NA
Lynch, 2012	CA184-041(a)	71	NA	NA	NA	NA	NA	NA	NA	NA	1.4	1.4
Reck, 2013	CA184-041(b)	42	2.4	2.4	2.4	2.4	NA	NA	NA	NA	0.0	0.0
Govindan, 2017	CA184-104	388	4.4	2.3	NA	NA	NA	NA	NA	NA	NA	NA
TOTAL			1.6 (1.3–2.0)	0.8 (0.6–1.1)	4.0 (3.3–4.8)	1.8 (1.3–2.4)	4.5 (3.9–5.1)	1.5 (1.2–1.9)	8.7 (7.8–9.6)	0.3 (0.2–0.5)	0.6 (0.3–1.0)	0.3 (0.1–0.6)

All 1: includes all Common Terms classified by Clinical Adverse Events (CTCAE) grades.

Serious2: includes CTCAE grades 3,4, or 5. NA: not available

**Figure 3:**
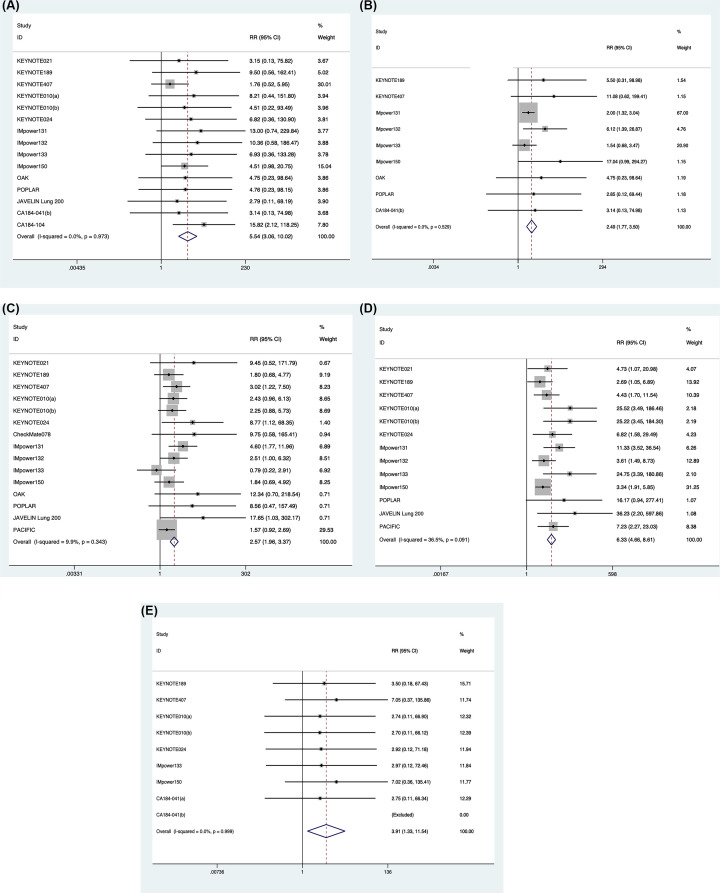
Forest plot of colitis (A), hepatitis (B), pneumonitis (C), hypothyroidism (D) and hypophysitis (E) in lung cancer patients treated with ICI versus chemotherapy

In the present study, compared with patients treated in control arms, those treated with ICI were at higher risk for IRAEs. Figure 3 showed that ICI therapy increased the frequency of immune-related colitis (RR: 5.54; 95%CI: 3.06–10.02; *P*<0.001), though events were rare. Patients treated with ICI drugs were at a higher risk for any grade hepatitis (RR: 2.49; 95%CI: 1.77–3.50; *P*<0.001) and pneumonitis (RR: 2.57; 95%CI: 1.96–3.37; *P*<0.001). Patients were more likely to experience hypothyroidism (RR: 6.33; 95%CI: 4.66–8.61; *P*<0.001) and hypophysitis (RR: 3.91; 95%CI: 1.33–11.54; *P*=0.013) compared with patients in the chemotherapy.

### Quality of included studies and sensitivity analysis

Risk of bias of the included RCTs was showed in Supplementary Table S1. Most studies have experienced low risk, especially the generation of random sequences. Unclear risk of bias was mainly focused on performance bias (blinding of participants and personnel). To examine the stability of the combined results, we conducted a sensitivity analysis after removing conference proceedings (Supplementary Figure S1). After these analyses, the results indicated that the outcome remained consistent.

## Discussion

Included more than 14,000 patients in advanced lung cancer, the present study was performed to analyze adverse events of ICI therapy versus the standard treatment to further our understanding of the safety of this emerging class of drugs. The pooled study indicated that ICI therapy was safer than chemotherapy, especially ICI monotherapy or anti-PD-1 drug in NSCLC. But ICI-chemotherapy increased the incidence of TRAEs, and anti-CTLA-4 antibodies was less safe in grade 3-5 TRAEs. Organ special IRAEs including colitis, hepatitis, pneumonitis, hypothyroidism and hypophysitis were uncommon but the risk was increased compared with control treatment.

Chemotherapy has always been the most commonly used class of antineoplastic drugs for advanced cancers. Traditional chemotherapeutic drugs work by killing rapidly dividing cells, whether they are tumor or healthy. Due to the long-term clinical application, the toxicities of chemotherapy drugs that reduce the quality of life of patients have been clearly demonstrated [47]. In the era of precision medicine, it is proposed that the treatment should not only cure diseases, but also restore patients’ health with the maximum quality of life [48]. Fortunately, the development of immunotherapy challenged the management of treatment-related toxic effects. Like the prior study, ICI drugs were overall less toxic than chemotherapy especially in monotherapy, and combining an ICI with chemotherapy increased the rate of grade 3 or worse severity TRAEs [49,50]. Although combined therapy resulted in significantly longer overall survival and progression-free survival than chemotherapy, its cytotoxicity also improved, which should not be underestimated [51]. Moreover, it is worth mentioning that ICI therapy was safer in risk of TRAEs for NSCLC patients, but less safe for SCLC. This may be due to the different pathogenesis of these two cancer types. In terms of drugs, anti-PD-1 antibodies had the best safety profile in lung cancer, which was consistent with previous conclusions [52]. Theoretically, PD-1 antibody can bind to PD-1 protein on T cells, thus blocking the binding of PD-1 to PD-L1 and PD-L2, while PD-L1 antibody can only interact with PD-L1, so it can only block the binding of PD-1 to PD-L1. All that meant PD-1 antibody emerged as the best option for treatment in advance lung cancer patients with greater survival condition and low incidence of TRAEs.

IRAEs represent the immune effect of incorrect stimulation of the immune system on normal tissues. Compared with the toxicities caused by conventional treatment, the IRAEs of ICI drugs have unique characteristics in organs involved, pathogenesis patterns and severity [53]. A number of randomized controlled trials were summarized the general situation of IRAEs, including skin, gastrointestinal, pulmonary, hepatic and endocrine toxicities [29,32,33,42]. The present study focused on five organs special IRAEs, including colitis, hepatitis, pneumonitis, hypothyroidism, and hypophysitis. For lung cancer, the most common organ specific IRAE was hypothyroidism which occurred 8.7%. The incidence of pneumonitis and hepatitis reached 4.5% and 4.0%, respectively. In addition, a recent study evaluated the risk of IRAEs in patients treated with anti-PD-1 and anti-PD-L1 drugs [14]. Our findings regarding risk of IRAEs were similar that those treated with ICI drugs were at higher risk for each organ specific adverse event compared with patients treated in control arms. Precise explanations for these observed differences were unknown, but this high-risk situation suggests that routine thyroid function tests and chest CT examinations should be added to patients with ICI therapy [54,55].

All the results emphasized a need for increased awareness and careful monitoring of patients with lung cancer during immunotherapy for the possibility of adverse events, particularly in IRAEs. Although most IRAEs are typically manageable with supportive treatment and glucocorticoids, uncommon fatal events have been reported increasingly [56–58]. The mechanism of IRAEs was still unclear. Health care providers need to maintain a high index of suspicion when patients develop worsening of symptoms and take appropriate measures to diagnose, initiate corticosteroids at the right time. Moreover, careful multidisciplinary consultation should be conducted in each case of suspected IRAEs to avoid improper disease management. In addition to advances in treatment strategies, identifying and improving the use of predictive biomarkers will also be critical for identifying patients most likely to benefit from treatment [59].

There were several limitations in the current study. First, the follow-up time was different from included studies (range 8–24 months), and the patients might have been discharged from hospital at the time of measurement. For example, it was reported that pneumonitis occur between 7.4 and 24.3 months after taking ICI drugs. [60] Thus the frequency of adverse events might be influenced by the confounding effect of time. Second, the methods for identifying adverse events had not standardized. Even in CTCAE, overlapping definitions confuse the recognizing of specific adverse events which leaded to potential uncertainty about data quality. As such, recognizing adverse events that usually depended on investigators’ evaluation might cause errors. A classic example was that immune-related colitis could be classified as colitis or diarrhea. Third, we combined all ICI drugs into experience group, including several therapeutic agents. However, because such a subdivision would result in more subgroups with smaller sample sizes, the cohorts were not subdivided according to these agents. Looking ahead, longer follow-up and special attention to various adverse events are needed to enhance our understanding.

## Conclusions

The present study indicated that immunotherapy was superior to chemotherapy in terms of safety profiles, especially ICI monotherapy. Patients treated with ICI therapy in NSCLC were less reported TRAEs than in SCLC. A lower risk of TRAEs was favored by anti-PD-1 antibodies over anti-PD-L1 antibodies and anti-CTLA-4 antibodies. Compared with standard treatments, ICI drugs increased the risk of organ-specific IRAEs, although the overall incidence remained low. For clinicians, it was important to monitor all IRAEs in lung cancer patients treated with ICI drugs.

## Supplementary Material

Supplementary Figure S1 and Appendix Table S1

## Abbreviations

ALK: anaplastic lymphoma kinase
CI: confidence interval
CTCAE: common terms classified by clinical adverse events
CTLA-4: cytotoxic T-lymphocyte-associated protein 4
EGFR: epidermal growth factor receptor
ES-SCLC: extensive-stage small-cell lung cancer
FDA: Food and Drug Administration
ICI: immune checkpoint inhibitor
IRAE: immune-related adverse event
KRAS: kirsten rat sarcoma
NSCLC: non–small-cell lung cancer
PD-1: programmed death 1
PD-L1: programmed death ligand 1
PD-L2: programmed death ligand 2
RCT: randomized controlled trial
ROS1: rat osteosarcoma
RR: risk ratio
SCLC: small-cell lung cancer
TRAE: treatment-related adverse event
