# Supplementary material for: Treatment- and immune-related adverse events of immune checkpoint inhibitors in advanced lung cancer
Source: Biosci Rep. 2020 May 7;40(5):BSR20192347. doi: 10.1042/BSR20192347 (PMC7953488; doi:10.1042/BSR20192347)
Supplement: Supplementary Figure S1 and Appendix Table S1 [file BSR-2019-2347_supp.pdf]

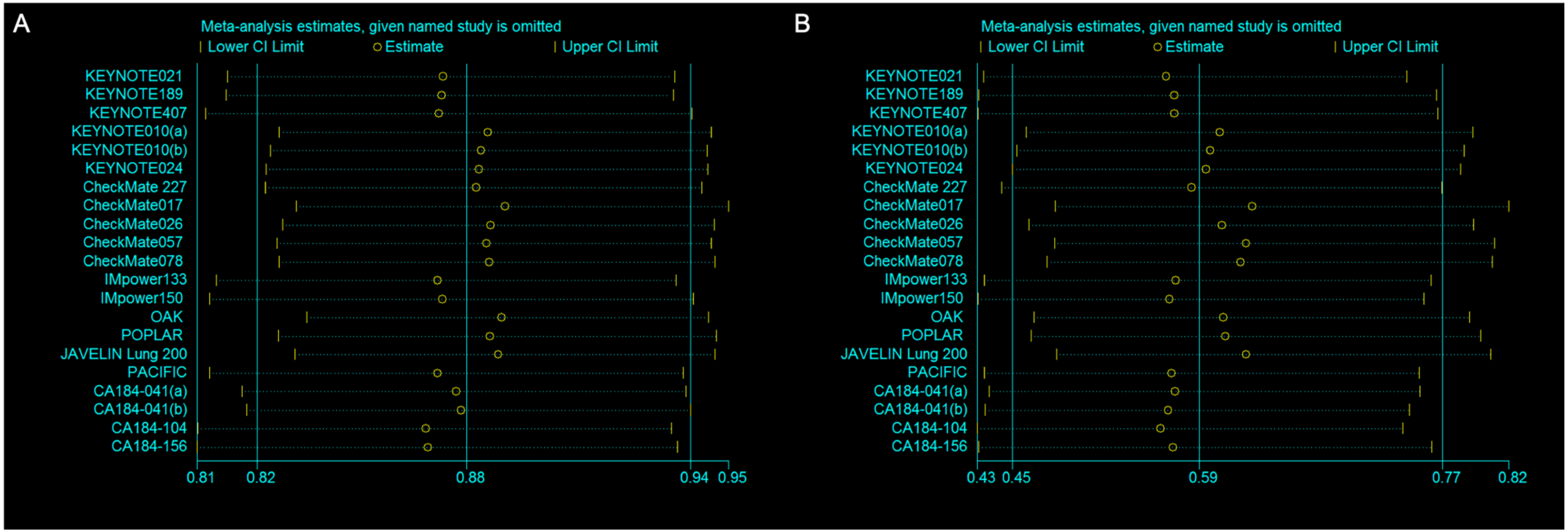

A
Meta-analysis estimates, given named study is omitted
Lower CI Limit
Estimate
Upper CI Limit
KEYNOTE021
KEYNOTE189
KEYNOTE407
KEYNOTE010(a)
KEYNOTE010(b)
KEYNOTE024
CheckMate 227
CheckMate017
CheckMate026
CheckMate057
CheckMate078
IMpower133
IMpower150
OAK
POPLAR
JAVELIN Lung 200
PACIFIC
CA184-041(a)
CA184-041(b)
CA184-104
CA184-156
0.81
0.82
0.88
0.94
0.95
B
Meta-analysis estimates, given named study is omitted
Lower CI Limit
Estimate
Upper CI Limit
KEYNOTE021
KEYNOTE189
KEYNOTE407
KEYNOTE010(a)
KEYNOTE010(b)
KEYNOTE024
CheckMate 227
CheckMate017
CheckMate026
CheckMate057
CheckMate078
IMpower133
IMpower150
OAK
POPLAR
JAVELIN Lung 200
PACIFIC
CA184-041(a)
CA184-041(b)
CA184-104
CA184-156
0.43
0.45
0.59
0.77
0.82

SupplementaryAppendix

TableS1 Qualityassessment: riskofbiasbyCochraneCollaboration'stool

StudyID	Randomsequencegeneration	Allocationconcealment	Blindingofparticipantsandpersonnel	Blindingofoutcomeassessment	Incompleteoutcomedata	Selectivereporting	Otherbias
**KEYNOTE021**	Low	Low	High	High	Low	Low	Low
**KEYNOTE189**	Low	Low	Low	Low	Low	Low	Low
**KEYNOTE407**	Low	Low	Low	Low	Low	Low	Low
**CheckMate227(a)**	Low	Low	Low	Low	Low	High	High
**IMpower131**	Low	Low	unclear	unclear	High	Low	High
**IMpower132**	Low	Low	unclear	unclear	High	Low	High
**IMpower133**	Low	Low	Low	Low	Low	Low	Low
**IMpower150**	Low	Low	High	High	Low	Low	Low
**PACIFIC**	Low	Low	Low	Low	Low	Low	Low
**CA184-041(a)**	Low	Low	Low	Low	High	High	Low
**CA184-041(b)**	Low	Low	Low	Low	High	High	Low
**CA184-104**	Low	Low	Low	Low	Low	Low	Low
**CA184-156**	Low	Low	Low	Low	Low	Low	Low
**KEYNOTE010**	Low	Low	High	High	Low	Low	Low
**KEYNOTE024**	Low	Low	High	High	High	High	Low
**KEYNOTE042**	Low	Low	unclear	unclear	High	Low	High

**CheckMate017**	Low	Low	High	High	Low	Low	Low
**CheckMate026**	Low	Low	High	High	Low	Low	Low
**CheckMate057**	Low	Low	High	High	Low	Low	Low
**CheckMate078**	Low	Low	High	High	Low	Low	Low
**CheckMate227(b)**	Low	Low	High	High	Low	Low	Low
**OAK**	Low	Low	High	High	High	High	Low
**POPLAR**	Low	Low	High	High	High	High	Low
**JAVELINLung200**	Low	Low	High	High	Low	Low	Low
